# The Impact of the Covid-19 Pandemic on Cleft Lip and Palate Service Delivery for New Families in the United Kingdom: Medical and Community Service Provider Perspectives

**DOI:** 10.1177/10556656221074870

**Published:** 2022-02-23

**Authors:** Danielle McWilliams, Bruna Costa, Sabrina Blighe, Marc C. Swan, Matthew Hotton, Nichola Hudson, Nicola Marie Stock

**Affiliations:** 1 Centre for Appearance Research, 1981 University of the West of England, Bristol, United Kingdom; 2 Spires Cleft Centre, 11269 John Radcliffe Hospital, Headley Way, Headington, Oxford, United Kingdom

**Keywords:** psychosocial development, ethics/health policies, cleft lip and/or palate, Covid-19, service delivery

## Abstract

**Objectives:**

Professionals in the United Kingdom providing care to new families affected by cleft lip and/or palate (CL/P) had to adapt to ensure families’ needs were met during a time of uncertainty due to Covid-19. The aims of this study were to explore the impacts of the pandemic on CL/P care provision for new families from the perspectives of professionals working in medical and community settings along with any personal impact on professionals and their reflections on the future of CL/P care.

**Design:**

Semistructured interviews (n  =  27) were completed about experiences from March 2020 to October 2020 with consultant cleft surgeons (n  =  15), lead clinical nurse specialists (n  =  8), and staff working at the Cleft Lip and Palate Association (n  =  4). Transcripts were analyzed using inductive thematic analysis.

**Results:**

Three themes were identified: (1) the impact of Covid-19 on the provision of cleft care in the United Kingdom, including working conditions, delays to treatment, and Covid-19 policies; (2) the impact of the pandemic on professionals’ mental health, including personal distress and concerns about Covid-19 exposure; and (3) reflections on the future of CL/P care, whereby professionals expressed both hope and concern about the Covid-19 recovery effort.

**Conclusions:**

The ongoing Covid-19 pandemic has impacted CL/P service delivery for new families significantly, warranting recommendations for cohesive psychological support for families in addition to a safe and resourced recovery effort. Support for professionals is also suggested, following existing evidence-based models for providers’ needs that address the difficulties of working throughout challenging times.

## Introduction

Infants born with a cleft lip and/or palate (CL/P) require multidisciplinary treatment throughout childhood and often beyond. In the United Kingdom, this care is provided free of charge at the point of use by the National Health Service (NHS). Given that a CL/P team referral is made as soon as a baby is born and this support is all provided on an NHS, evidence-based pathway, seeking treatment on a private basis is rare in the United Kingdom. The NHS treatment pathway begins with a prenatal or postnatal referral to a specialist NHS CL/P team following a diagnosis and is lifelong. Aside from primary surgeries, CL/P treatment for new families also involves outpatient appointments and input from nonsurgical members of the multidisciplinary treatment team (MDT), including clinical psychologists, clinical nurse specialists, speech and language therapists, and geneticists as indicated.^1,2^ In particular, the clinical nurse specialist plays a key role in a new families’ care, supporting them through diagnosis, early navigation of feeding, initial MDT appointments, and primary surgery.^3^ Alongside NHS provision for CL/P care, the Cleft Lip and Palate Association (CLAPA) is a registered UK charity serving everyone impacted by CL/P, from prenatal support for parents through to adults looking to return to treatment. CLAPA's services typically include confidential one-to-one and group support, regular social and fundraising events, community-informed information, a feeding service supplying specialist bottles, and online social communities.^4^

As a result of the Covid-19 pandemic, health care provision and services were severely disrupted worldwide. In August 2020, the World Health Organisation (WHO) published a report stating that 90% of participating countries worldwide (n  =  129) reported interruptions to essential health care services, with 66% reporting cancellations to elective services, which can include CL/P surgeries. Redeployment of staff, government restrictions on travel, insufficient personal protective equipment, and changes in policies surrounding treatment were also key contributors to Covid-19-related service disruption (World Health Organization).^5^ In the United Kingdom specifically, a government-enforced lockdown of all face-to-face public services and a ban on nonessential travel was introduced in March 2020 in an attempt to control the spread of the virus and minimize the demands placed on NHS resources.^6^

Surgery across all CL/P services was stopped for several months after the lockdown announcement, until new guidelines for surgeons were introduced in July 2020, 4 months later (Federation of Surgical Specialty Associations).^7^ Four priority levels for restarting surgery within the NHS were set, with cleft palate surgery at ‘Category 3’ (to avoid breaching the recommended age of 13 months) and cleft lip surgery at ‘Category 4’ (considered low priority, which can wait indefinitely). Covid-19 restrictions on infection control and social distancing also required operating rooms and clinics to operate at vastly reduced capacities, which only exacerbated delays further.^8^ In addition to delays to surgery, the UK-wide lockdown resulted in suspension of face-to-face outpatient appointments and home visits across the majority of health services. This meant that appointments were either canceled or had to be conducted via videoconferencing or telemedicine platforms, including within CL/P services (World Health Organization, 2020).^9^ Across private settings, capacity was also vastly reduced as a result of an agreement reached between NHS England and the Independent Healthcare Providers Network, such that even private beds and resources would be allocated to Covid-19 patients where needed.^10^

The impact of Covid-19 reached beyond health care services and into the work of charitable organizations. In the case of CL/P, the lockdown required many CLAPA staff to be placed on temporary leave. In addition, fundraising events were canceled, and face-to-face services had to cease with little notice. In parallel, demand for charitable support increased exponentially; a paradox that was later dubbed “the perfect storm” for charities.^11^

The Covid-19 pandemic and the impact of subsequent restrictions imposed by Government have resulted in unprecedented disruption to CL/P care in the United Kingdom and globally. The aim of the present study was to explore the impact of the early stages of the pandemic (from March 2020 to October 2020) on CL/P professionals and to explore their perspectives on how services for new families had changed or been affected.

## Method

### Ethical Considerations

Ethical approval was granted by the Faculty Research Ethics Committee at (*university, city*). The ethical guidelines of the British Psychological Society were also followed.

### Design

This study employed an inductive, qualitative approach. Individual, one-to-one interviews were carried out with consultant CL/P surgeons (surgeons), clinical nurse specialists (nurses), and staff working at CLAPA during the first “wave” (March-July 2020) of the Covid-19 pandemic. A semistructured interview schedule was created, informed by recent literature about the impact of the pandemic on health care services (World Health Organization, 2020), as well as prior knowledge about optimal CL/P service delivery (eg, Ref. ^12^). Input from leading specialist health professionals (including surgeons, nurses, and clinical psychologists) was also sought and their feedback was incorporated into the final version of the interview schedule. Table 1 provides a more detailed overview of the interview schedule.

**Table 1.** Summary of the interview schedule for surgeons, nurses and CLAPA staff.

Demographic information	*For surgeons and nurses:*
	Cleft team
	Team context (eg, how many surgeons/nurses on the team)
	Years since qualifying
	Years working in UK cleft services
	*For CLAPA staff:*
	Current job role
	Years working for CLAPA
Impact of Covid-19 on service delivery	*For surgeons and nurses:*
	Current status of services in the region
	Which services stopped and which kept running
	Differences in treatment for new families prior to and during lockdown
	Expected time needed to “catch up” with primary surgeries
	Impact of pandemic on service audit and research
	*For CLAPA staff:*
	Current status of community support
	Which services stopped and which kept running
	Differences in support provision for families prior to and during lockdown
Management of service/treatment delay	*For surgeons and nurses:*
	How decisions are made about surgery and treatment provision and by whom
	Communication with and reactions from families regarding treatment delays
	*For CLAPA staff:*
	Number of staff placed on temporary leave and how these decisions were made
	How changes in services have been communicated to staff and families
	Reactions from families about changes to community service provision
Impact of Covid-19 on families and staff	*For all:*
	Perceived impacts of lockdown on new families
	Perceived impacts of treatment delays on other members of the CL/P team
	Projected impact of treatment delays on child development
	Strategies implemented/planned to mitigate impact on families and team members
	Personal impacts of Covid-19 on interviewee
Future service delivery	*For surgeons and nurses:*
	Projected long-term changes to surgical protocols
	*For all:*
	Prevention of negative impacts in the case of an additional “wave”
	New ways of working that could become permanent
	Hopes and concerns for the future of health care and community CL/P services
	Any positive consequences and key learning points

Abbreviations: CLAPA, Cleft Lip and Palate Association; CL/P, cleft lip and/or palate.

All questions were open-ended and the interviewers were able to prompt interviewees to provide more details where appropriate. Interviews were conducted between August and October 2020 by the first, second, and senior authors, all of whom are trained in qualitative methods. Interviews were carried out using Microsoft Teams or via telephone, ranging from 33 to 114 min, and lasted ∼52 min for nurses, 60 min for surgeons, and 58 min for CLAPA staff (with an overall mean of 57 min).

### Procedure

Potential participants were initially approached through the surgery (n  =  36) and nursing (n  =  28) UK Cleft Clinical Excellence Networks (CENs). A CEN is composed of all specialist clinicians working within a particular discipline and field; in this case, nurses and surgeons working in CL/P care. Additional follow-up emails were used where necessary. CLAPA staff were recruited directly via email.

Potential participants who expressed an interest in the study were screened for eligibility. Inclusion criteria stipulated that participants had to be working in UK CL/P services prior to and during the first “wave” of the pandemic. Staff who were currently or who had recently been on temporary enforced leave were excluded. Eligible participants were sent a participant information sheet containing further details about what participation in the study would entail and key ethical information, such as confidentiality and their right to withdraw. Participants who agreed to take part were asked to provide verbal consent prior to the interview commencing. The interviews were audio-recorded and transcribed verbatim by the first, second, and third authors. All eligible participants who volunteered during the recruitment period were interviewed and data were collected until no new information was forthcoming.

### Data Analysis

Interview transcripts were analyzed using inductive thematic analysis (TA; Braun and Clarke).^13^ As per Braun and Clarke's 6 prescribed stages of TA, the first author became familiar with the data (1) and generated initial codes (2). Codes were then collated into themes (3). The first, second, and third authors reviewed the themes and discussed them with the senior author (4). Last, final themes were defined and named (5), and the manuscript was produced (6).

## Results

### Participants

In total, 27 professionals participated, constituting nurses (n  =  8), surgeons (n  =  15), and CLAPA staff (n  =  4). All nurses in the sample were female and had between 6 to 24 years of experience in the CL/P service, with a mean of 15 years. Of the surgeons who participated, 11 were male and 4 were female. Surgeons had spent between 1 and 24 years working in CL/P care, with a mean of 11 years. Collectively, participants represented 10 out of the 12 specialist CL/P networks in the United Kingdom and the Republic of Ireland.

Four members of CLAPA staff were specifically selected for their positions in the organization, length of service, and breadth of knowledge. The majority (n  =  3) of participants were female. Participants had worked for CLAPA for between 2.5 and 9 years, with a mean of 5 years.

### TA Findings

The analysis identified 3 themes: (1) the impact of Covid-19 on the provision of cleft care in the United Kingdom; (2) the impact of the pandemic on professionals’ mental health; and (3) professionals’ reflections on the future of CL/P care. Themes are presented below alongside exemplar quotes. Shortened quotations are indicated by […] and additions for the purpose of clarity are indicated by (…). For transparency, guidelines around quantifying language have been adhered to (Hill et al.)^14^. For example, “all” refers to all or all but one, “most” refers to more than half, and “some” refers to less than half but more than 2.

### Theme 1: The Impact of Covid-19 on the Provision of Cleft Care in the United Kingdom

The first theme concerns the changes that UK CL/P teams were required to make throughout the first “wave” of the pandemic. Across the 3 participant groups, discussions took place around what the service looked like as restrictions began coming into force (March 2020) and again at the time of interview (August-October 2020). Participants reported that although broad guidance on the provision of health care was issued, some hospitals had more clinical capacity and were therefore more able to adapt more quickly to the restrictions imposed.

(The advice was that) children aren’t seriously affected by coronavirus, […but] the impact of me not doing surgery was going to be significant. So we decided to start operating again [as soon as possible], so I was lucky.—Surgeon.

In comparison, most hospitals had to close the entire CL/P service down to make way for the huge surge in patients with coronavirus.

We were anticipating huge demand in our Intensive Care Unit. We lost an awful lot of […] space, time, and staff, so we had no capacity to run normal theatres. There was no elective operating for a long time.—Surgeon.

Participants were aware that in light of the indefinite pause on NHS CL/P surgery, some families had decided to pay for their child's primary surgeries privately. Most participants raised concerns about who would be accountable for the aftercare and how to realign these families with the NHS CL/P treatment pathway later on.

That is definitely a concern because (families) don’t get the MDT follow-up (when they go private). They’ll get the surgery but generally not a lot either side.—Nurse.

However, some surgeons expressed a willingness to operate privately on those families who can afford it, with the aim of shortening NHS waiting lists for those who cannot.

We would consider it. It wouldn't happen within NHS time, and …we would only do it for our own patients anyway, so it would [reduce] the waiting list and get us back up to speed more quickly.—Surgeon.

Once CL/P surgery was able to restart, hospitals had to work around the changes in protocols that were necessary to adhere to Covid-19 restrictions. One of these was that most CL/P networks required babies and families to self-isolate for up to 14 days prior to coming into hospital. Some CL/P services had found this fairly easy to manage:

We did ask (families) to self-isolate, although they were naturally doing that themselves (during lockdown) anyway […]. The Government advice is very similar to what we’re saying, (so) I’ve found that most parents have not argued.—Nurse.

Conversely, some participants were concerned that the new surgical protocols were arduous for some families, especially those from low socioeconomic backgrounds:

It was OK if you had a generally affluent family that were working from home and it was easy for them to self-isolate for 14 days […]. The tricky ones were the working-class families that were living in multiple occupancy households […], so you had the mother and baby isolating at home but actually, what else was going on with that household?—Nurse.

In-person nurse outreach appointments for new families were also stopped for several months, resulting in consultations being carried out online and a reduction in overall contact between patients and CL/P teams.

If a baby was born with a cleft in the maternity unit we could only do video calls, which missed out 21 babies […]. Some of them we (still) haven’t actually met.—Nurse.

Participants also observed that more general support that would typically be offered to new parents, including in-person support from midwives and health visitors (responsible for the general monitoring of infant and parent health and well-being) had dramatically reduced. This left some CL/P nurses feeling they had to compensate by providing general health advice as well as advice related to CL/P.

There was no community feeding support. There were no community helpers to weigh the [babies], (and) limited community midwifery support. So, we were having to do a lot more telephone and video calls with families just trying to meet their general needs as new parents.—Nurse.

For many months, community support from CLAPA was also impacted significantly, with all in-person events being canceled. CLAPA staff were also aware of the lack of midwifery support and health visiting provision that new families were receiving and put considerable effort into ensuring their feeding service was able to continue running.

We cancelled all of our events…(but) it was actually quite a nice thing, that we could say reassuringly to people: “no matter what happens, come rain or shine, we’re still going to be able to deliver these bottles.”—CLAPA staff.

Finally, reduced capacity, both in terms of staffing and clinical environments, often meant that research ceased and audit policies were breached, although most participants felt clinical activities should take priority.

At this stage, [audit is] not a priority… We’re not seeing our children that need to be seen, so why are we putting research and audit before the routine stuff?—Nurse.

### Theme 2: The Impact of the Pandemic on Professionals’ Mental Health

The second theme focuses on the impact of the pandemic on participants’ mental health in both a professional and personal context. Most participants spoke of their individual circumstances outside of work and how this contributed to the overall stress they were experiencing.

There was a lot of worry about people getting ill and dying, and their relatives getting ill and dying, […and] that had an impact on everybody.—Surgeon.

I haven’t hugged my [adult] son for six months.—Surgeon

For those participants who continued to work in a hospital setting, the reality of the pandemic and increasing death rates was visible on a daily basis.

At the hospital we had a mega-mortuary planted on one of our car parks… I cried quite a lot that day, because it felt like the whole world was going to die.—Nurse.

Walking around the hospital…it was eerie… You walk into a hospital and expect to see people, [but] it was just empty.—Nurse.

The circumstances that led to changes in service delivery also led to changes in the working conditions of health professionals and CLAPA staff. Reports of stress, increased workload, and heightened work pressure were common.

I felt like everything that I was in charge of was suddenly two, three, four times more important… The first month or two was just an absolute blur of logistically trying to make stuff work.—CLAPA staff.

Most participants reported feeling powerless, as the process for allocating surgery slots was taken out of their hands.

I don’t think we were particularly helped by the [surgical] prioritisation [process]. Someone, the night before, had to write down the criteria, and that person was not a cleft surgeon.—Surgeon.

We feel like we are in front of our families, swords drawn, trying to get into theatre, and the (management) are kind of keeping us out because they’re prioritising other things… I owe more to my patients than I do to the (management).—Surgeon.

The increase in work pressure, alongside the need to navigate restrictions and changing protocols, led to tension between some health professionals, both within their teams and with other CL/P colleagues across the United Kingdom.

I really wanted the cleft service for the whole of the UK to have a far more cohesive and unified position nationally, and I thought that was lacking.—Surgeon.

Our clinical director made all the decisions and there was a lot of disagreement with the surgeons about it. (They) became quite autocratic about it.—Nurse.

A strong feeling of uncertainty and the stress that came with this was present across all interviews. Participants were often left frustrated and unable to relieve families of their anxieties, as they themselves could not predict how circumstances would change week to week.

I had no idea what to tell [families], because no one had any idea what to tell them.—CLAPA staff.

These children don’t have a plan. There is no “we’re getting to you in four months.” We don’t know when we’re going to get to them […]. Not knowing I think is causing an ongoing trauma to those families.—Surgeon.

The restrictions on CL/P services resulted in participants reporting distress at not being able to carry out their professional duties and provide their patients with the best care possible.

If a family phones with a concern, we would usually just jump out and see (them) at home, and it was difficult to change that state of mind. We’re not providing as a good a service as we want to be.—Nurse.

Every Thursday evening the neighbours will be out on the doorstep, clapping away (as a gesture of appreciation for the NHS) and…we haven’t done very much.—Surgeon.

CLAPA staff were also personally impacted by their inability to provide a comprehensive service, which was compounded by the added pressure of having to maintain fundraising efforts to keep the charity afloat.

We can’t meet [families’] needs, and at the same time, we have to ask them for all of this money. That was difficult… I would have felt better about it if we had all of these incredible resources to give them, but we didn’t.—CLAPA staff.

### Theme 3: Professionals’ Reflections on the Future of CL/P Care

The third theme describes the various adaptations that were made to CL/P care in the United Kingdom during the first “wave” of the pandemic and participants’ thoughts, hopes, and concerns about the future of CL/P services. The suspension of surgery caused delays across all CL/P teams and prompted the introduction of new prioritization guidelines. Under these guidelines, many primary CL/P operations did not take place within the timeframe that the usual UK pathway would advise. This caused much concern to participants, particularly in the case of cleft lip surgery, which fell under Category 4.

Some of the [cleft lip surgeries] we’ve stopped, and put on the back burner, but how long can you do that for? There’s still a need for them to be done. So how long do you wait?—Surgeon.

Participants were also deeply concerned about the long-term impact of Covid-19-related service changes on new families’ well-being.

There may well be some deeper emotional scars left by those early days. Sometimes if (families have) really struggled in those first couple of months it stays with them for a long time.—Nurse.

At the time of interview, all CL/P teams were conducting remote consultations to some degree. There was a spectrum of opinions on the efficacy and longevity of virtual appointments, ranging from a feeling that remote contact with families is impractical and impersonal, to a belief that virtual appointments can make the service more efficient and more accessible.

You have to see people in person, … to examine them, but also the holistic aspect and understanding how they feel and what questions they want to ask. I think it would be a great loss [to adopt video appointments permanently].—Surgeon.

Some families quite enjoyed the fact that mum and dad could be in the sitting room having a cup of coffee, and if we were 10 minutes late, so be it. But we did try to keep to time, much more than we would in person.—Surgeon.

CLAPA staff also highlighted positive consequences of the move to online services and were looking to employ a hybrid model of online and in-person events in the future.

I think this [experience] has really helped us to look outside the box a little bit more and instead of always doing the tried and tested things, it's showing us that actually, we can experiment and it’s not too difficult, and it’s not too risky… It is easier to reach increased numbers remotely and we are reaching new people.—CLAPA staff.

For some participants, the pandemic had created more team camaraderie and a greater appreciation for colleagues. This often arose from the understanding that navigating the pandemic has been a notable ordeal for the entire CL/P community.

[This experience has] solidified our roles, it’s created a respect across everybody. I think people have realised what everybody does and I think we’re all level, we’re all the same… We’re more together even though we’re apart.—Nurse.

Yes, surgery is hugely important, but actually it’s having a team that work together… We all held things together.—Surgeon.

Nonetheless, concerns regarding how CL/P services would be commissioned and funded in future were common.

I am concerned that people will look at [the service changes]…and say, “Do we really need a specialist nurse? Can we manage virtually?” And would services be cut on the back of that?—Nurse.

Funding is a really big concern. We just can’t do most of the [in-person] community fundraising activities…which is where most of our money comes from… We already work on such a knife edge.—CLAPA staff.

Finally, participants reflected on any further impacts that further waves of Covid-19 might bring. Most participants were highly anxious about how the CL/P service would cope if further restrictions were imposed.

It’s going to be very difficult for us to catch up, particularly if further waves are going to happen. One can have virtual clinics and virtual consultations, but you can’t have virtual surgery… It does worry me that it's going to be a long journey.—Surgeon.

However, others felt that having made it through the first “wave,” they would already have strategies in place and be more prepared in future.

If something was to happen again, I think having gone through this process, of being forced into putting things online […], we’d be in a good place to be doing things quite quickly.—CLAPA staff.

I can’t see us grinding to a halt and ending up with zero surgery for as long a period of time again. So, what we’re trying to do is get ahead enough so that if it happens again, we will still be OK.—Surgeon.

## Discussion

The Covid-19 pandemic has impacted health care systems, charitable organizations, and the staff working within them around the world. The aim of the present study was to explore the impact of the pandemic on CL/P services for new families from the perspective of specialist CL/P professionals working within medical and community settings in the United Kingdom. Interviews with surgeons, nurses, and CLAPA staff offered important insight into the restrictions imposed on CL/P services, the attempts of service providers to adapt to these restrictions, and the impacts of the pandemic on professionals’ well-being. In addition, the future of CL/P services was discussed and key learning points were extracted, which are summarized and displayed in Table 2.

**Table 2.** Summary of findings and key learning points from the present study.

Theme identified	Summary of findings	Key learning points
Theme 1: *The Impact of Covid-19 on the Provision of Cleft Care in the UK*	Some cleft lip and/or palate (CL/P) teams were better equipped to cope with Covid-19-related restrictions than others Some families had chosen to pay privately for their child's primary CL/P operation(s) New surgical protocols require families to self-isolate and test for Covid-19 prior to treatment commencing Community-based support was considerably reduced All professionals reported concern about the delays in CL/P care for new families and about what long-term impacts this would have In most CL/P teams, both research and audit ceased in favor of clinical priorities	The pandemic has highlighted existing inequities across regions The increase in private treatment may cause longer term challenges for follow-up Some families may struggle to adhere to these additional protocols CL/P professionals had to go above and beyond to ensure a basic level of community-based support was offered Psychological screening and support may be needed for families who have been impacted by the delays to treatment Reducing the long-term physical and psychological impact of delayed CL/P care on new families should be a priority Without ongoing research and audit, the community could lose valuable information about the impacts of the pandemic
Theme 2: *The Impact of the Pandemic on Professionals’ Mental Health*	Professionals were exposed to a high level of personal and professional distress Professionals reported a considerable degree of “moral injury” at not being able to deliver an optimal service	Opportunities for psychological screening and support for professionals should be a key consideration of the recovery effort A tiered system of moral injury prevention and intervention is indicated
Theme 3: *Professionals’ Reflections on the Future of CL/P Care*	Professionals were concerned about newly imposed guidelines stipulating that CL/P surgeries were “low” priority Remote contact with families can improve access and attendance, but can also impose unwanted barriers The pandemic created more team camaraderie and a greater appreciation for colleagues Funding was a key future concern among professionals Professionals were concerned about the further impacts of an additional “wave” of the pandemic	The psychological impact of treatment delays on families should be taken into consideration, and efforts to “catch up” on waiting lists should be supported A hybrid approach to CL/P appointments may offer a helpful solution in future Personal growth, resilience, and improved team connections can arise from challenging situations if managed well Ongoing efforts are needed to evidence the rationale for continued services There is a need for CL/P service providers to implement adaptive strategies and to prepare for the possibility of future service disruption

### Supporting the Psychological Well-Being of Professions Working in CL/P Services

The ongoing Covid-19 pandemic has resulted in huge pressures on NHS resources and a reliance on staff to adjust. NHS CL/P services were closed down for 4 months and later restarted under strict restrictions, resulting in long treatment delays and significant disruption to routine care. CLAPA saw the majority of their staff being placed on temporary leave at the same time as the demand for support from families increased considerably. As a result of these sudden changes in protocol, participants in the current study cited high levels of stress, an increased workload, heightened work pressures, and tension between colleagues. In addition, the ongoing personal threat of Covid-19 to the health of family, friends, and colleagues was constant and particularly visible to those working in hospital settings. In the broader literature, as many as 23% of surveyed frontline NHS health care professionals met the clinical threshold for posttraumatic stress disorder in 2020.^15,16^ A further 47% met the criteria for clinical levels of anxiety and 47% met the clinical threshold for depression.^16^ These figures are higher than the rates recorded prepandemic, of 4%, 6%, and 3%, respectively.^17^ High levels of anxiety have also been reported among NHS staff regarding the risk of contracting Covid-19 and passing it on to other patients, colleagues, or loved ones.^18^ For those professionals placed on temporary leave, the impacts may also include long-term changes in individuals’ perceptions of their ability, motivation, and overall job security, also leading to comparisons about who is “more” or “less” valuable to an organization.^19^ The current study adds to the growing body of literature examining the impact that the pandemic has had on individuals and teams working to keep NHS services running and the crucial importance of the protection of staff well-being.

Participants in the current study also described a sense of disempowerment over the way families were being cared for and clear distress over not being able to carry out their normal roles and provide an optimal service. Participants were acutely aware of the known impacts of having a baby with CL/P on families,^20^^-^^23^ as well as the additional distress families were experiencing due to the pandemic (Costa et al.).^24^ Yet, participants were no longer able to provide families with any certainty regarding infants’ treatment trajectory, nor could they offer in-person practical and emotional support to ease families’ distress. Consequently, some professionals felt they were undeserving of the widespread public displays of appreciation for the NHS that were commonplace in shops, on streets, and throughout the media at the time, increasing feelings of frustration and inadequacy. In the wider literature, Covid-19 has triggered many reports of “moral injury” from NHS staff.^25,26^ Moral injury refers to the “profound psychological distress which results from actions, or the lack of them, which violate one's moral or ethical code.”^27^ A report by Tracy et al^28^ called for a tiered system of moral injury intervention, whereby primary prevention includes self-help resources, clear leadership, and full transparency from management, as well as efforts to maintain team camaraderie. NHS Staff Resilience Hubs providing access to mental health support services have been established since the current study interview period.^29^ However, during interviews, participants reported isolation from and tension between colleagues, as well as disappointment with the way decisions about CL/P services had been handled. Further, the secondary and tertiary tiers of the proposed moral injury intervention include full screening and linking to mental health services. This is particularly relevant to staff who are at risk of mental health concerns and/or moral injury as a result of Covid-19,^28^ although this targeted moral injury support did not seem to be explicitly available to the participants in the current study at the time of interview. Research has demonstrated that personal growth, resilience, and improved team connections can arise from challenging situations if managed well.^30^ This was reflected in participants’ narratives whereby greater team camaraderie and a greater sense of understanding and respect were mentioned as a result of working together through such a challenging period of time. Moving forward, opportunities for psychological screening and dedicated support for professionals should be a key consideration of the recovery effort.

### Determining the Future of Cleft Care

In the early 2000s, following a significant review of CL/P care in the United Kingdom,^31^ CL/P services underwent a period of centralization, reducing the number of hospitals providing care and implementing specialist standardized protocols across newly formed teams. These protocols included recommendations for the care of new families and the timeliness of primary surgery, which have since been shown to improve a range of family and patient outcomes, in addition to establishing a national process for audit and research.^12^ Recent cohort studies conducted in Denmark and Sweden concur that delayed palate surgery (at 36 months of age) results in poorer speech outcomes, which require more extensive speech therapy, compared with those who had their surgery within the timeframes indicated by the UK pathway (by 12 months of age.^32^ The arrival of the Covid-19 pandemic disrupted these standard UK protocols, with many families subsequently experiencing a much reduced and delayed service to the one that has been in place for 2 decades. There is a growing concern among CL/P professionals that delaying cleft lip operations indefinitely as “category 4” will have long-term negative impacts on child psychological adjustment, particularly in relation to social stigma relating to a child having a visibly open cleft lip.^33^ Long-term monitoring of the impact of service disruption and surgical delays caused by the pandemic is needed. However, much of the UK's audit and research processes were forced to cease at the height of the first “wave.” Efforts to reinstate this important work should be supported where possible, since without research and audit, the community will lose valuable information pertinent to the well-being of the affected cohort and the lessons to be learned for future service delivery.

In response to the unprecedented disruptions to CL/P services, NHS teams and CLAPA staff had to adapt quickly, with most contact with families being diverted online. Although initially unfamiliar, participants reported that virtual contact with families had improved attendance and accessibility, with families being able to speak with teams and engage with CLAPA from the comfort of their own homes, avoiding the expense and time taken to travel to in-person appointments and events. Nonetheless, participants felt that delivering certain aspects of care online was ineffective, particularly in cases where feeding support was needed, and/or if families reported chaotic home lives. Some professionals were concerned that virtual contact would become the “norm” and would replace in-person visits and events. These findings are in line with broader research exploring the use of telehealth. For example, a large qualitative study reported that in general, remote appointments were most acceptable to patients when a prior relationship with the health professional had already been established through in-person visits.^34^ This may be particularly relevant for CL/P care, where the importance of the relationship between the clinical nurse specialist and the family for optimal care is well understood.^3,35^ Issues with delivering CL/P care online also include difficulties with speech assessments due to sound and image quality and maintenance of orthodontic treatment.^9^ Similarly, and given the conflicting emotions parents often experience around the time of the primary surgery,^36^ it would not be an ideal prospect for parents to only meet their child's surgeon on the day of the surgery. Overall, a hybrid approach may be most appropriate in future, with a return to in-person support when it is safe to do so, while keeping an online platform open for appointments and events where it is appropriate and/or preferred.

At the time of interview, the first national “lockdown” had ended, and restrictions were beginning to ease over the summer months. However, guidelines for surgical prioritization and changes to hospital protocols were still in effect. Many participants were concerned about their team's ability to “catch up” on their surgical waiting lists with these restrictions still in place, particularly if an additional “wave” of the pandemic were to occur. While the interviews predominantly focused on the impact of the pandemic on CL/P care for new families, professionals also commented on delays to other elective surgeries, including those most often performed in late adolescence/early adulthood (eg, orthognathic surgery, rhinoplasty, and lip revision). Participants were concerned that other adult services, such as those recently developed by CLAPA, would also slip down the priority list. Findings from this study indicated that all professionals within CL/P care provision are working tirelessly to advocate for families and make up for the delays that have been necessary to make way for the Covid-19 effort, but there is a long way to go. Moving forward, preparations need to be made to support professionals to return to a timely, consistent, and accessible service for families impacted by CL/P.

## Methodological Considerations

The current study offers new insight into the impact of the first “wave” (March-October 2020) of the Covid-19 pandemic on UK CL/P care from the perspective of specialist NHS health professionals and members of CLAPA staff, in addition to key learning points for the future. Since these interviews were conducted, a further 2 national “lockdowns” have been imposed in the United Kingdom, and cases have continued to rise with the discovery of new Covid-19 variants, despite a successful vaccination program. It may be assumed that the impacts identified in the current study have continued, increased, and/or changed in the time that has since passed. Further work to elicit the perspectives of professionals at this stage in the pandemic would be useful, as would quantitative studies to determine the impact of Covid-19 on key outcome statistics and audit figures. Future exploration of the impact of the pandemic on the rest of the treatment pathway is also warranted.

In the current study, 2 of the 12 CL/P networks in the United Kingdom and the Republic of Ireland were not represented. Equally, resources would not allow for all CL/P disciplines to be interviewed. Instead, this study focused on the perspectives of those most often involved in the early care of new families. However, clinical psychologists were consulted during the design and write-up of this study. While this article has provided valuable insight, it should be noted that not all possible viewpoints were captured.

## Conclusions

The NHS continues to face significant pressures relating to the Covid-19 pandemic. This study offers insight into how the pandemic has impacted CL/P service delivery and the well-being of staff involved in supporting new families embarking on the CL/P treatment pathway. As part of the recovery effort, commissioners and management teams should remain cognizant of the evidence base behind the CL/P treatment pathway, such that care can resume to agreed standards. Staff need access to time, space, and funding to facilitate this, in addition to emotional support and an acknowledgment of the stress and significant pressure that working during the pandemic has created.
